# A receptor-like protein RMC is involved in regulation of iron acquisition in rice

**DOI:** 10.1093/jxb/ert290

**Published:** 2013-09-07

**Authors:** An Yang, Yansu Li, Yunyun Xu, Wen-Hao Zhang

**Affiliations:** ^1^State Key Laboratory of Vegetation and Environmental Change, Institute of Botany, Chinese Academy of Sciences, Beijing 100093, PR China; ^2^The Institute of Vegetables and Flowers, Chinese Academy of Agricultural Sciences, Beijing 100081, PR China; ^3^Key Laboratory of Plant Molecular Physiology, Institute of Botany, Chinese Academy of Sciences, Beijing 100093, PR China; ^4^Research Network of Global Change Biology, Beijing Institutes of Life Science, Chinese Academy of Sciences, Beijing, PR China

**Keywords:** Fe concentration, iron deficiency, *OsRMC*, rice *(Oryza sativa)*, root system, seed Fe concentration.

## Abstract

Iron (Fe) is one of the essential mineral elements for plant growth and development. Acquisition of Fe by plants is mediated by a complex network involving Fe mobilization, uptake by root cells, and transport within plants. Here, we evaluated the role of a previously clarified gene encoding a receptor-like protein from rice, *OsRMC*, in the regulation of Fe acquisition by comparing Fe concentration, biomass, and expression patterns of genes associated with Fe mobilization and transport in wild-type (WT) rice with those in *OsRMC* overexpression and RNA interference (RNAi) knockdown transgenic rice plants. Expression of *OsRMC* was upregulated in both shoots and roots upon exposure of WT to Fe-deficient medium. Expression levels of *OsRMC* were positively correlated with Fe concentration in rice plants under both Fe-sufficient and Fe-deficient conditions such that overexpression and RNAi lines had higher and lower Fe concentration in both roots and shoots than WT plants, respectively. Moreover, overexpression of *OsRMC* conferred greater accumulation of Fe in mature seeds under Fe-sufficient conditions. *OsRMC* may also play a role in regulation of Fe deficiency-induced changes in root growth, as evidenced by greater and smaller root systems of *OsRMC* overexpression lines and RNAi lines than WT under Fe-deficient conditions, respectively. Several Fe deficiency-responsive genes including *OsDMAS1*, *OsNAS1*, *OsNAS2*, *OsNAAT1*, *OsIRT1*, *OsYSL15*, and *OsIRO2* were up- and downregulated in *OsRMC*-overexpressing and RNAi plants compared with WT rice plants. These novel findings highlight an important role of *OsRMC* played in mediation of Fe acquisition and root growth in rice, particularly under Fe-deficient conditions.

## Introduction

Mineral deficiency is the most widespread dietary problem, affecting more than half the world’s population, particularly in developing countries (Lee *et al.*, 2009). Rice is a major crop and primary food source in many countries, and thus increasing its mineral content is an effective way to improve the nutritional quality of plants (Lee and An, 2009). Among the mineral elements, iron (Fe) is one of the essential nutrients for plant growth and human health (Curie and Briat, 2003; Lee *et al.*, 2009). Fe deficiency results in chlorosis and reduced crop yield and quality. Deficiency in bioavailable Fe in food affects a large proportion of the world population. For instance, anaemia due to deficiency in Fe nutrition is one of the most serious problems in humans (Hell and Stephan, 2003; Lee *et al.*, 2009).

Although Fe is the second most abundant metal element in the earth’s crust, its bioavailability to plants is often limited, especially in neutral-to-alkaline soils where Fe occurs largely in the form of insoluble hydroxides and oxides (Guerinot and Yi, 1994; Romera and Alcántara, 2004). To cope with Fe deficiency in these soils, plants have evolved two distinct strategies to mobilize and acquire Fe, classified as strategy I in non-graminaceous monocots and dicots, and strategy II in graminaceous monocots (Marschner *et al.*, 1986; Mori, 1999). In strategy I plants, Fe^3+^ is reduced to Fe^2+^ and then transported into root cells by an Fe^2+^ transporter, IRON-REGULATED TRANSPORTER 1 (IRT1) (Eide *et al.*, 1996; Robinson *et al.*, 1999; Vert *et al.*, 2002). In contrast, in strategy II graminaceous plants, acquisition of Fe is involved in exudation of phytosiderophores, Fe^3+^ chelators of the mugineic acid (MA) family (Takagi *et al.*, 1984; Inoue *et al.*, 2009; Lee *et al.*, 2012). In addition to uptake of Fe^3+^, rice plants can also uptake Fe^2+^ via IRT transporters in the waterlogged paddy field conditions where Fe^2+^ is abundant because of the low redox potential (Ishimaru *et al.*, 2006).

Exudation of MAs is an important step for acquisition of Fe by strategy II plants. MAs are synthesized from *S*-adenosyl-l-methionine catalysed by three sequential enzymes: nicotianamine (NA) synthase (NAS), nicotianamine aminotransferase (NAAT), and deoxymugineic acid synthase (DMAS) (Inoue *et al.*, 2003; Bashir *et al.*, 2006; Lee *et al.*, 2012). In rice, genes encoding these enzymes have been isolated (Inoue *et al.*, 2003, 2008; Bashir *et al.*, 2006). Among them, expression of *OsNAS1*, *OsNAS2*, and *OsNAS3* that encode rice NAS is differentially regulated by Fe deficiency (Inoue *et al.*, 2003). Bashir *et al.* (2006) reported that expression of *OsDMAS1* is upregulated under Fe-deficient conditions in shoots and roots. *OsYSL15* is responsible for the acquisition of Fe^3+^-MA from the rhizosphere and strongly upregulated by Fe deficiency (Inoue *et al.*, 2009; Lee *et al.*, 2009). OsIRT1, an Fe^2+^ transporter, is expressed mainly in roots and induced under Fe-limited conditions (Ishimaru *et al.*, 2006). In addition, several transcription factors have been identified to play a role in regulation of Fe homeostasis. These include OsIDEF1 (Kobayashi *et al.*, 2007, 2009, 2012), OsIDEF2 (Ogo *et al.*, 2008), OsIRO2 (Ogo *et al.*, 2007), OsIRO3 (Zheng *et al.*, 2010), and OsbHLH133 (Wang *et al.*, 2013). However, the overall signalling networks associated with Fe-deficiency responses in plants in general and in rice in particular remain largely to be dissected.

The receptor-like kinases are characterized by an N-terminal hydrophobic signal peptide, and an internal hydrophobic sequence followed by a basic ‘stop-transfer’ sequence (Hardie, 1999). These receptors, which are localized at the cell surface, are often involved in sensing signals associated with developmental, environmental, and hormonal cues, and with transducing the signals to trigger adaptive responses (Torii, 2000; Morris and Walker, 2003; Afzal *et al.*, 2008). *OsRMC* is a member of the domain unknown function 26 (DUF26) (cysteine-rich repeat) subfamily (Jiang *et al.*, 2007). Knockdown of the *OsRMC* gene by RNA interference (RNAi) led to altered root development by targeting jasmonic acid (JA) signalling (Jiang *et al.*, 2007). Zhang *et al.* (2009) reported that RNAi knockdown of *OsRMC* in transgenic rice makes the transgenic rice plants more tolerant to salt stress, implying that *OsRMC* plays a role in regulation of the plant response to salt stress. Moreover, a recent study reported that the expression of a putative *OsRMC* is downregulated by phosphorus deficiency using a comparative proteome approach (Torabi *et al.*, 2009). However, there have been no detailed physiological studies to evaluate the role of *OsRMC* in regulation of mineral nutrients in plants so far. In the present study, we demonstrated that *OsRMC* positively regulated Fe acquisition in rice plants, such that overexpression and knockdown of *OsRMC* led to an enhanced and suppressed Fe accumulation in roots, shoots, and seeds, respectively. We further explored the physiological mechanisms by which the transgenic rice plants with altered expression levels of *OsRMC* differed from wild-type (WT) plants in terms of their responses to Fe deficiency.

## Materials and methods

### Plant materials and growth conditions

Rice plants (*Oryza sativa* L. ssp. *japonica*, cv. Zhonghua 10) were used as WT controls relative to the transgenic plants (overexpression and knockdown lines of *OsRMC* by RNAi, OE3, Ri1, and Ri4) in physiological experiments. The transgenic seeds used in the present study were kindly provided by Professor Kang Chong (Jiang, *et al.*, 2007). Seeds of WT and transgenic lines were germinated in dark at 28 °C for 3 d. Thereafter, the rice seedlings were pre-cultured hydroponically in one-half strength Kimura B solution containing 0.37mM (NH_4_)_2_SO_4_, 0.18mM KH_2_PO_4_, 0.18mM KNO_3_, 0.55mM MgSO_4_.H_2_O, 0.09mM K_2_SO_4_, 0.37mM Ca(NO_3_)_2_, 46.2×10^–3^ mM HBO_3_, 3.2×10^–4^ mM CuSO_4_, 7.7×10^–4^ mM ZnSO_4_, 9.1×10^–3^ mM MnCl_2_^.^4H_2_O, 3.6×10^–4^ mM H_2_MoO_4_, 0.70mM NaSiO_4_.9H_2_O, and 10.0×10^–2^ mM EDTA-Fe in a growth room with a 16h light (30 °C)/8h dark (22 °C) photoperiod, and the relative humidity was controlled at ~70%. The solution was refreshed every 3 d.

For analysis of *OsRMC* expression in transgenic plants, 2-week-old rice seedlings grown hydroponically in normal conditions were harvested. To determine the time course of Fe deficiency-induced changes in gene expression, 2-week-old rice seedlings grown in the control solution with a sufficient supply of Fe (100 μM EDTA-Fe) were transferred to Fe-deficient solution by omitting EDTA-Fe. To minimize the supply of Fe from seed endosperm, the seed endosperm was removed prior to transferring of rice seedlings to Fe-deficient medium. Shoots and roots were harvested for RNA extraction after exposure of rice seedlings to Fe-deficient medium for varying periods (0, 6, 12, 24, 72, and 168h). To determine the effect of deprivation of other mineral nutrients, including nitrogen (N), phosphate (P), potassium (K), and sulfur (S) on *OsRMC* expression, 2-week-old rice seedlings grown in the control solution with a sufficient supply of nutrients were transferred to solutions containing no nitrogen (–N), phosphate (–P), potassium (–K), and sulfur (–S), respectively. Plants were harvested for RNA extraction after the treatments for 5 d. To investigate the phenotypes of the transgenic rice plants with overexpression and knockdown of *OsRMC* and WT rice plants, 1-week-old seedlings were germinated and grown in both Fe-sufficient (100 μM EDTA-Fe) and Fe-deficient (0 μM EDTA-Fe) medium. After 15 d of treatment, height, shoot biomass, and root biomass were measured. Shoots and roots were dried for 3 d at 80 °C before being weighed.

### Measurement of metal content

To determine Fe content in the transgenic and WT rice plants, elemental analysis was conducted on seedlings grown under both Fe-sufficient and Fe-deficient conditions. Shoot and root samples were harvested, ground to a fine powder, and digested in 6ml of concentrated nitric acid and 2ml of hydrogen peroxide with a CEM MARS system. Total metal contents were determined by inductively coupled plasma mass spectrometry. To determine the concentrations of metals in seeds of WT and transgenic plants, seeds that were harvested over the previous 3 years were used to measure the content of Fe, Zn, Mn, and Cu. WT and transgenic plants were grown in a paddy field located at the Institute of Botany, Chinese Academy of Sciences.

### Measurement of chlorophyll (CHL) content

To determine CHL content in both WT and transgenic rice plants, leaves were harvested, weighed, and extracted with aqueous ethanol (95% v/v) overnight. Newly formed leaves were weighed and then ground with aqueous acetone (80% v/v) and centrifuged at 10 000*g* for 5min. Absorbance (*A*) readings of the supernatant were recorded at wavelengths of 646 and 665nm. Total CHL content was calculated as 7.18*A*_665_+17.32*A*_646_, and was expressed as μg CHL mg^–1^ of fresh weight.

### Measurement of root length and surface area

Roots of WT and transgenic rice seedlings grown in both Fe-sufficient and Fe-deficient medium for 15 d were scanned with a digital scanner (Expression 10000XL; Epson, Japan) and analysed with WinRHIZO/WinFOLIA software (Regent Instruments, Canada). The total lengths of root and root surface area were obtained from the scanned root data. For measurements of primary root length, total length of the three longest adventitious roots, and number of adventitious roots, seeds were germinated in the dark at 28 °C for 3 d and grown in Fe-sufficient medium for 1 week. The seedlings were then grown hydroponically for 8 d in Fe-sufficient or Fe-deficient medium, and the plants were sampled for measurements.

### RNA isolation and real-time RT-PCR

Total RNA was isolated from leaves and roots using RNAiso reagent (Takara) and was reverse-transcribed into first-strand cDNA with a PrimeScript^®^ RT Reagent kit with PrimeScript^®^ RT reagent kit). Real-time PCR was performed in an optical 96-well plate with an Applied Biosystems Stepone^TM^ Real-Time PCR system. Each reaction contained 7.5 μl of 2× SYBR Green Master Mix reagent, 0.5 μl of cDNA samples, and 0.6 μl of 10 μM gene-specific primers in a final volume of 15 μl. The thermal cycle used was as follows: 95 °C for 10min, and 40 cycles of 95 °C for 30 s, 60 °C for 30 s, and 72 °C for 30 s. The following primers were used: *OsRMC*, 5′-TCGGAGGTGTACCCGTTCTACA-3′ and 5′-ACTCTTAATT TGTGCCATTTTATTCTAGCT-3′ (Zhang *et al.*, 2009); *OsNAS1*, 5′-GTCTAACAGCCGGACGATCGAAAGG-3′ and 5′-TTTCTC ACTGTCATACACAGATGGC-3′; *OsNAS2*, 5′-TGAGTGCGTG CATAGTAATCCTGGC-3′ and 5′-CAGACGGTGACAAACACC TCTTGC-3′ (Inoue *et al.*, 2003); *OsIRT1*, 5′-CGTCTTCTTCTTC TCCACCACGAC-3′ and 5′-GCAGCTGATGATCGAGTCTGAC C-3′; *OsDMSA1*, 5′-TCTCCTTCGCCACCATCCCTC-3′ and 5′-CC TTGCTCGTACACCCACCTCA-3′; *OsNAAT1* 5′-AGACCAGG CTACCCAAACTATG-3′ and 5′-CACCTCTTTGATAGCGATC C-3′; *OsYSL15*, 5′-ACTGGTACCCTGCAAACATAC-3′ and 5′-GC AATGATGCTTAGCAAGAAG-3′; *OsIRO2*, 5′-CTCCCATCGTT TCGGCTACCT-3′ and 5′-GCTGGGCACTCCTCGTTGATC-3′; and *actin*, 5′-ACCACAGGTATTGTGTTGGACTC-3′ and 5′-AG AGCATATCCTTCATAGATGGG-3′. Relative quantification (ΔΔ*C*_t_ method) was used to evaluate quantitative variation between the replicates. Amplification of *actin* (GenBank accession no. AB047313) was used as an internal control to normalize data. The expression level of genes in the WT under Fe-sufficient conditions was defined as 1.

### Statistical analysis of data

Analysis of variance was conducted with all samples compared with WT grown with different levels of Fe supply. Significant differences were evaluated using Student’s *t*-test when *P≤*0.05.

## Results

### Expression profiles of *OsRMC* under conditions of different nutrient deficiencies

**Fig. 1.**
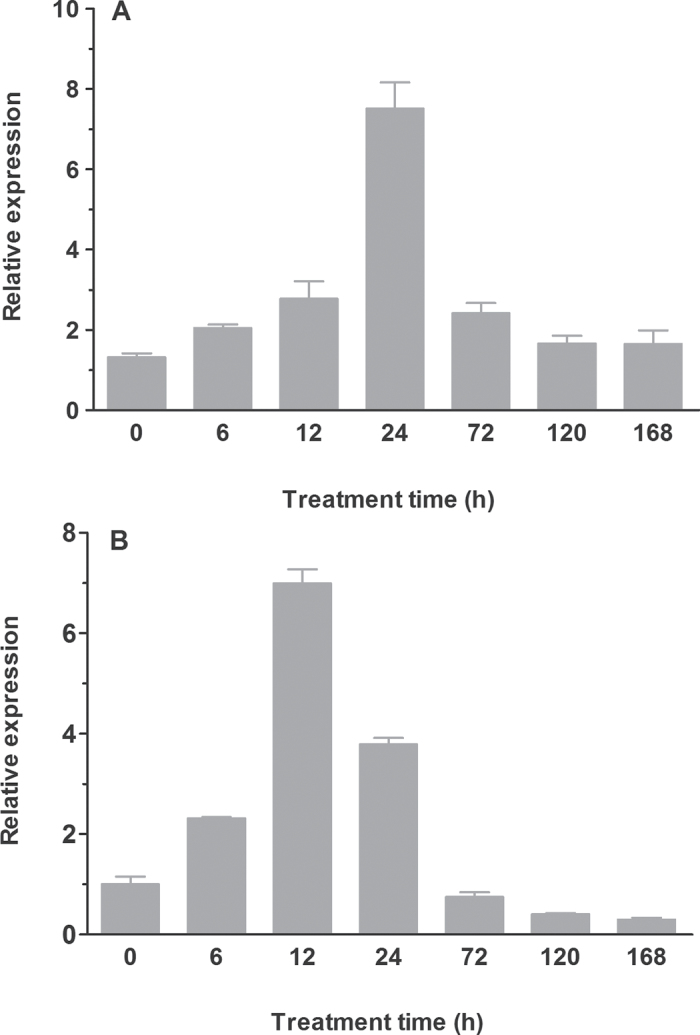
Expression patterns of *OsRMC* in rice. Time course of *OsRMC* expression under Fe-limited condition in shoot (A) and root (B). Total RNAs were prepared from 14-d-old seedlings of WT rice after the above treatment and then reverse transcribed. The resultant cDNAs were used as templates for real-time PCR, and *actin* was used as an internal control. Error bars are based on three biological replicates.

A gene, *OsRMC*, which encodes a receptor-like protein, was isolated in rice by Jiang *et al.* (2007). *OsRMC* has been shown to be involved in the regulation of JA-dependent root development and tolerance of rice plants to salt stress (Jiang *et al.*, 2007; Zhang *et al.*, 2009). To evaluate the role of *OsRMC* in Fe acquisition in rice, the responsiveness of *OsRMC* to Fe deficiency was studied. Upregulation of *OsRMC* expression in both shoots and roots was observed upon exposure of WT rice seedlings to Fe-deficient medium (Fig. 1). For instance, an increase in *OsRMC* transcripts in the shoot was detected after 6h of exposure to Fe-deficient medium, and the increase was more evident with prolonged exposure to Fe-deficient medium (Fig. 1A). Expression of *OsRMC* peaked by 24h of Fe-deficient treatment, and declined thereafter (Fig. 1A). A similar Fe deficiency-induced expression pattern of *OsRMC* in roots was observed (Fig. 1B). In addition, the expression of *OsRMC* was also upregulated by deprivation of P, K, and S, but not by N starvation (Supplementary Fig. S1 at *JXB* online).

### Expression levels of *OsRMC* in transgenic lines

**Fig. 2.**
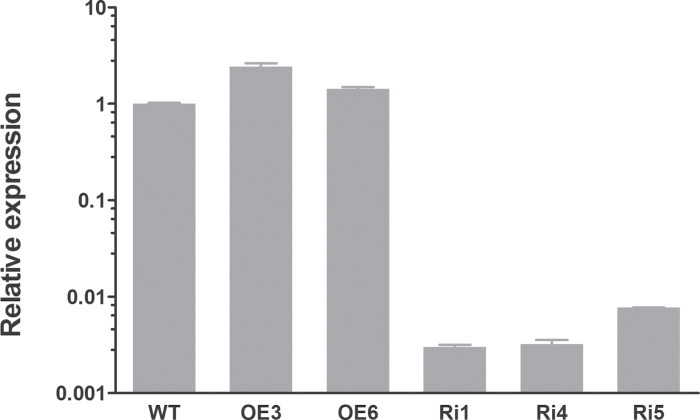
Expression levels of *OsRMC* in WT and transgenic rice. Total RNAs were prepared from the 14-d-old seedlings of the WT and transgenic rice and then reverse transcribed. The resultant cDNAs were used as templates for real-time PCR, and *actin* was used as an internal control. Error bars are based on three biological replicates.

The expression levels of *OsRMC*-overexpressing and RNAi knockdown lines were examined by real-time RT-PCR. Expression of *OsRMC* was enhanced in the overexpression lines (OE3 and OE6) and was suppressed significantly in the knockdown lines (Ri1, Ri4, and Ri5) (Fig. 2). One overexpression line (OE3) and two knockdown lines (Ri1 and Ri4) were chosen based on their expression levels of *OsRMC* and their seed availability to study the physiological function of *OsRMC* using T5 generations of these transgenic plants as well as WT rice plants.

### Transgenic rice plants exhibit a different response to Fe deficiency from WT plants

**Fig. 3.**
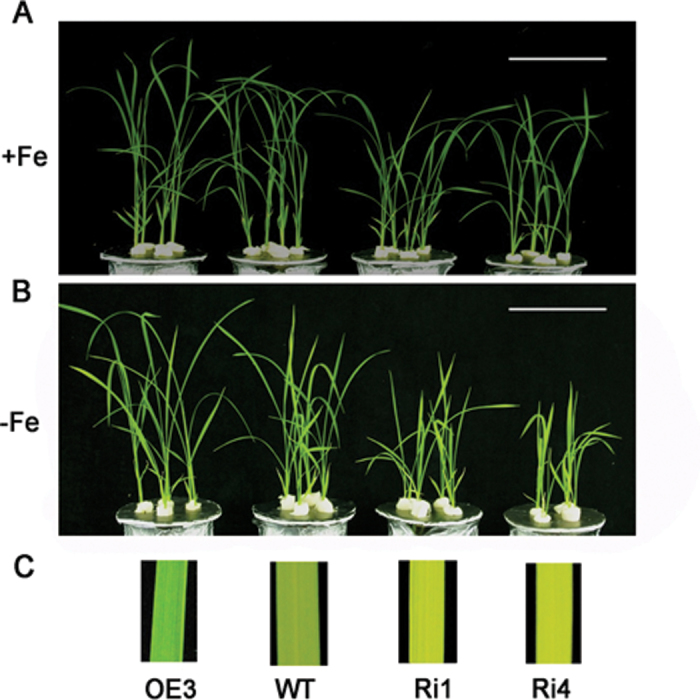
Growth performance of WT and transgenic rice plants grown at different Fe levels. (A) Growth performance of WT and transgenic rice plants grown under Fe-sufficient (A) or Fe-deficient (B) conditions. One-week-old rice seedlings grown in normal culture solution were transferred to culture solution supplied with 100 μM and without Fe in the greenhouse for 15 d. Bars, 10cm. (C) Photographs of chlorotic leaves after the –Fe treatment.

The observation that expression of *OsRMC* was induced by Fe deficiency prompted us to examine whether the transgenic rice plants with altered expression levels of *OsRMC* differed from their WT counterpart in response to Fe deficiency. As shown in Fig. 3A, no evident differences in phenotypes between WT and the Os*RMC*-overexpressing line OE3 were observed when grown in Fe-sufficient medium. However, the overexpressing line OE3 exhibited greater growth than WT plants when grown in Fe-deficient medium (Fig. 3B, C). Accordingly, the overexpressing line and WT plants had comparable shoot and root biomass when grown in Fe-sufficient medium (Table 1). By contrast, the shoot and root biomass of the two RNAi lines was significantly lower than that of WT plants under Fe-sufficient conditions (Table 1). In addition to biomass, overexpression of *OsRMC* led to a higher height of transgenic plants than WT plants under Fe-deficient conditions, while WT and OE3 plants did not differ in their height under Fe-sufficient conditions (Table 1). In contrast, the plant height of the two RNAi lines was significantly shorter than that of WT and the overexpression line OE3 under both Fe-sufficient and Fe-deficient conditions (Table 1).

**Table 1.** Plant height, dry shoot biomass, dry root biomass, and CHL content of WT and transgenic plantsOne-week-old seedlings were grown hydroponically for 15 d in Fe-sufficient (100 μM EDTA-Fe) or Fe-deficient (0 μM EDTA-Fe) medium were sampled for measurements. Data are means ±SD of three independent experiments. Asterisks (*) indicate significant differences at *P*<0.05 with regard to WT by Student’s *t*-test. DW, dry weight; FW, fresh weight.

Genotype	Shoot biomass (mg DW per plant)	Root biomass (mg DW per plant)	Plant height (cm)	CHL content (μg mg^–1^ FW)
Fe-sufficient medium				
WT	47.42±3.45	7.45±1.23	25.78±2.23	3.34±0.14
OE3	45.58±4.73	7.68±1.33	24.56±2.18	3.36±0.22
Ri1	30.79±3.13*	5.47±1.17*	20.24±1.01*	2.79±0.43*
Ri4	29.57±2.46*	5.28±1.37*	20.04±1.17*	2.98±0.16*
Fe-deficient medium				
WT	30.13±1.41	5.34±1.23	18.13±0.89	0.80±0.13
OE3	34.61±1.37*	6.10±0.98	20.68±0.32*	1.47±0.21*
Ri1	16.34±1.78*	3.98±1.44*	12.13±1.65*	0.34±0.22*
Ri4	15.78±1.59*	3.76±0.61*	11.43±0.78*	0.31±0.12*

No differences in CHL content between WT and the overexpressing line OE3 were observed under Fe-sufficient conditions, while the RNAi lines had lower CHL contents (Table 1). A marked reduction in CHL content in both WT and the transgenic rice plants was observed when grown in Fe-deficient medium (Table 1). However, the Fe deficiency-induced reduction in CHL content was less and greater in overexpressing and RNAi lines, respectively, than in WT plants, leading to higher and lower CHL content in the overexpression and RNAi lines, respectively, than in WT plants under Fe-deficient conditions.

### Overexpression and suppression of *OsRMC* makes transgenic rice plants more and less efficient, respectively, at acquiring Fe than WT plants

**Fig. 4.**
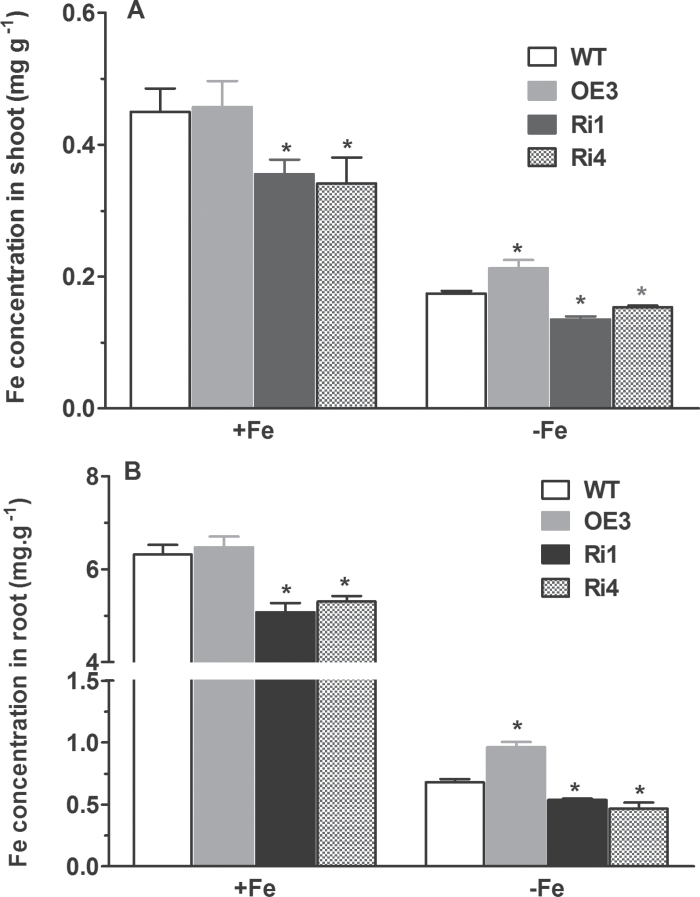
Quantification of metal content in shoots, roots, and seeds of WT and transgenic plants. (A) Shoot tissue. (B) Root tissue. One-week-old seedlings were grown hydroponically for 15 d in Fe-sufficient or Fe-deficient medium and the plants were sampled for measurements. Significant differences from WT were determined by Student’s *t*-test (*P<0.05).

The observations that transgenic rice plants overexpressing *OsRMC* exhibited greater biomass, a greater height, and less reduced CHL contents than WT plants under Fe-deficient conditions suggested that the expression level of *OsRMC* might be positively correlated with tolerance of rice plants to Fe deficiency. To test this hypothesis, we measured Fe concentrations in both shoots and roots of rice plants at the seedling stage. Under Fe-sufficient conditions, Fe concentrations in the *OsRMC*-overexpressing plants were marginally, but statistically insignificantly, higher than those in WT. However, *OsRMC*-overexpressing plants accumulated a greater amount of Fe than WT plants under Fe-deficient conditions (Fig. 4). In contrast to the overexpression line, Fe concentrations in RNAi lines were much less than WT plants grown under the same conditions (Fig. 4). In addition to Fe, we also compared other divalent metal concentrations in the transgenic plants with those in WT plants under both Fe-sufficient and Fe-deficient conditions. Among the metals examined, Cu concentrations in shoots of the two RNAi lines were significantly lower than in WT plants under both Fe-sufficient and Fe-deficient conditions, while the OE3 line had a higher Cu concentration in shoots than in WT plants under Fe-deficient conditions (Fig. S2 at *JXB* online). In contrast, no significant differences in concentrations of Mn and Zn in both shoots and roots of the transgenic plants and WT under both Fe-sufficient and Fe-deficient conditions were observed (Fig. S2).

### Transgenic rice plants with altered expression of *OsRMC* and WT plants exhibit different root phenotypes

**Fig. 5.**
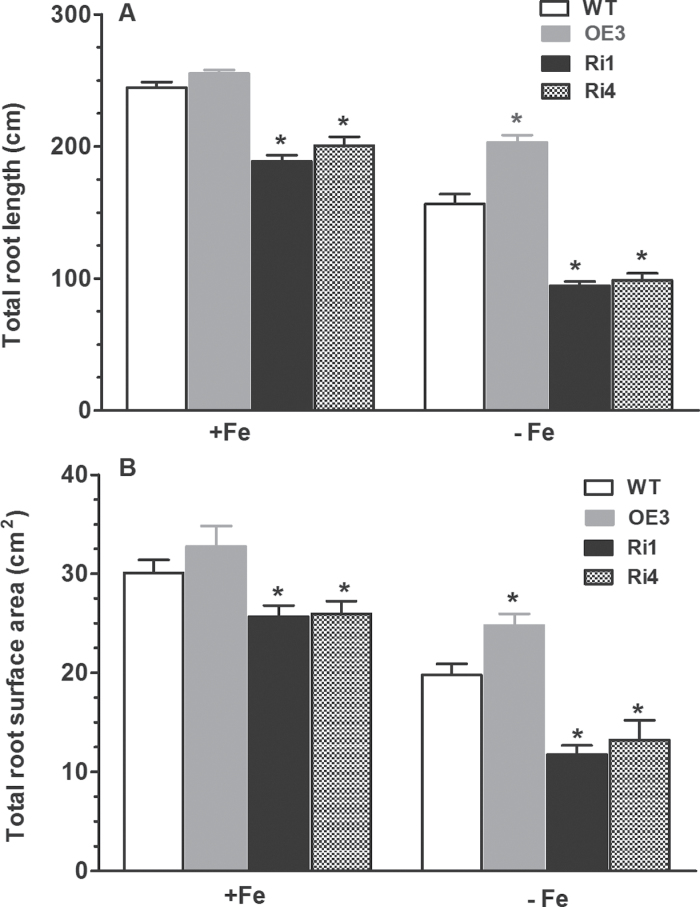
Root parameters of WT and transgenic plants from solution culture experiments. (A) Total root length. (B) Root surface area. One-week-old seedlings were grown hydroponically for 15 d in Fe-sufficient or Fe-deficient medium and plants were sampled for measurements. Data are means ±SEM (*n*=8). Significant differences from WT were determined by Student’s *t*-test (*P<0.05).

**Fig. 6.**
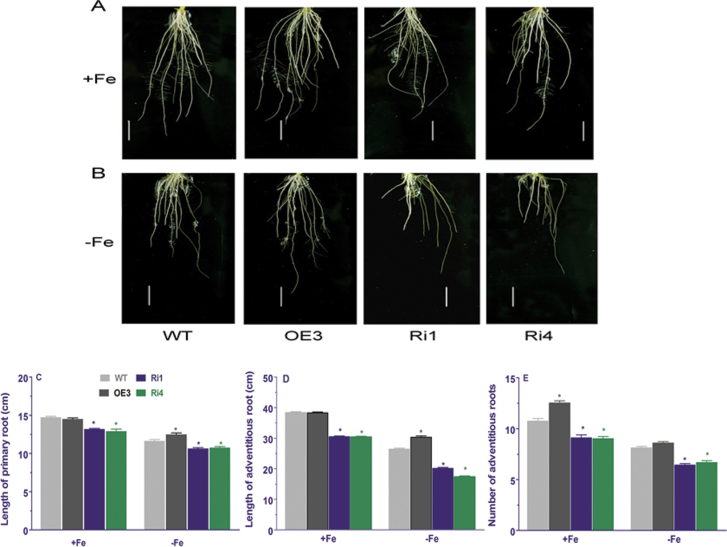
(A, B) Root phenotypes of 1-week-old WT and transgenic rice plants grown in Fe-sufficient (A) and Fe-deficient medium (B) for 8 d. (C–E) Primary root length (C), total length of three longest adventitious roots (D), and number of adventitious roots (E) for WT and transgenic rice plants grown in Fe-sufficient and Fe-deficient medium were measured after exposure to Fe-sufficient and Fe-deficient medium for 8 d. Significant differences from WT were determined by Student’s *t*-test (*P<0.05). Bars, 2cm.

Root phenotype is an important trait for plants to efficiently utilize Fe under Fe-deficient conditions. Under Fe-deficient conditions, the total root length and root surface area of the *OsRMC*-overexpressing rice seedlings were significantly higher than those of WT seedlings (Fig. 5). In contrast, knockdown of *OsRMC* by RNAi in the transgenic rice plants reduced root growth compared with WT plants grown in Fe-sufficient and Fe-deficient medium, as evidenced by the significantly reduced total length and surface areas of RNAi plants compared with WT plants (Fig. 5). In addition, the primary root length and total length of the three longest adventitious roots as well as number of adventitious roots in WT, OE, and RNAi plants were measured. Under Fe-sufficient conditions, no significant differences were observed in primary root length and total length of the three longest adventitious roots between WT and the *OsRMC*-overexpressing rice seedlings (Fig. 6). However, the number of adventitious roots in OE and RNAi plants was greater and less than in WT plants, respectively, under Fe-sufficient conditions (Fig. 6). Knockdown of *OsRMC* by RNAi led to the shorter primary root length, total root length of the three longest adventitious roots and less adventitious root number compared with WT plants under both Fe-sufficient and Fe-deficient conditions (Fig. 6). These results indicated that disruption of *OsRMC* expression may alter root development under both Fe-sufficient and Fe-deficient conditions. The large root systems can facilitate Fe acquisition by increasing root surface area for Fe uptake and exploring more soil space.

### Overexpression of *OsRMC* results in greater accumulation of Fe in rice seeds

**Fig. 7.**
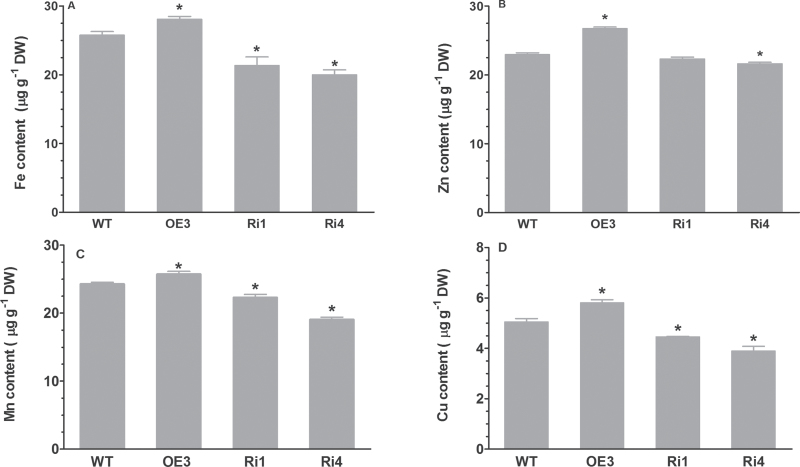
Concentrations of Fe (A), Zn (B), Mn (C), and Cu (D) in WT and transgenic seeds. Seeds harvested over the last 3 years were used to measure the level of metal content. WT and transgenic plants were grown in a paddy field located at the Institute of Botany, Chinese Academy of Science. The field tests were conducted three times. Data are means ±SD. Significant differences from WT were determined by Student’s *t*-test (*P<0.05).

In addition to in shoots and roots, the content of Fe and other mineral nutrients in mature seeds of WT and overexpressing and RNAi lines was also determined. Seeds of the OE3 line contained a greater amount of Fe than those of WT seeds (Fig. 7A). In contrast, Fe contents in the RNAi seeds were significantly less than in WT seeds (Fig. 7A). For instance, the Fe content in seeds of the overexpressing line OE3 was 8.9% higher than that in WT seeds, while the Fe content in seeds of the RNAi line Ri1 was 17.1% lower than in WT seeds. In addition to Fe content, the Zn content in the OE3 seeds was also significantly higher than that in WT seeds (Fig. 7B). In contrast, the Zn content in seeds of the two RNAi lines was reduced compared with those in WT seeds (Fig. 7B). Compared with WT and RNAi grains, the contents of Mn and Cu were higher in overexpressing plants than in WT plants (Fig. 7C, D).

### Expression of Fe deficiency-responsive genes is altered in transgenic rice plants

**Fig. 8.**
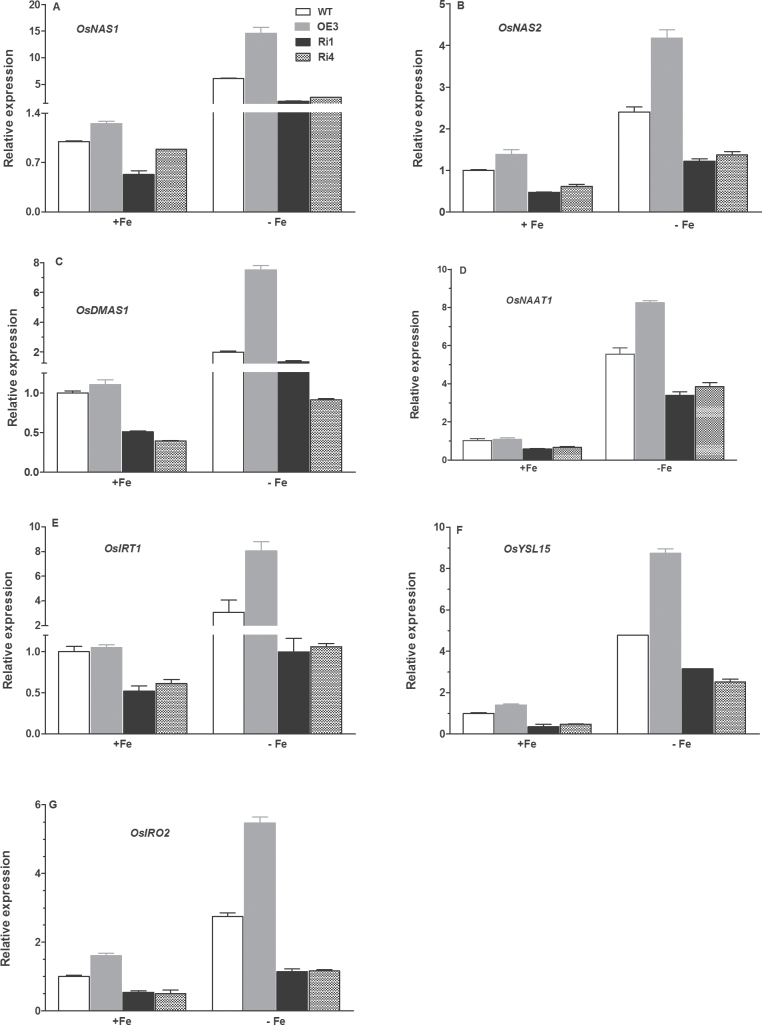
Expression patterns of some Fe deficiency-responsive genes in the roots of transgenic and WT plants. The following genes were analysed: *OsNAS1* (A), *OsNAS2* (B), *OsDMAS1* (C), *OsNAAT1* (D), *OsIRT1* (E), *OsYSL1* (F), and (G) *OsIRO2*. Total RNA was extracted from 2-week-old rice seedlings grown under control and Fe-limited stress conditions for 24h. Transcript levels were measured by real-time PCR. *Actin* was used as an internal control. Error bars are calculated based on three biological replicates.

Given the important role of MA in Fe acquisition in strategy II rice plants (Takagi, 1976; Takagi *et al.*, 1984), we monitored the changes in transcript levels for the genes involved in MA biosynthesis in WT and transgenic rice plants grown in Fe-sufficient and Fe-deficient medium. These include genes encoding nicotianamine synthase (*OsNAS1*, *OsNAS2*), nicotianamine aminotransferase (*OsNAAT1*) and deoxymugineic acid synthase (*OsDMAS1*). In addition to biosynthesis of MAs, the effect of *OsRMC* on expression patterns of *OsIRT1* and *OsYSL15* encoding transporters of Fe^2+^ ions (Ishimaru *et al.*, 2006) and Fe^3+^-MA (Inoue *et al.*, 2009) in rice, respectively, was evaluated. We also examined the role of OsIRO2, which is an Fe-responsive transcription factor involved in regulation of Fe homeostasis (Ogo *et al.*, 2007), in OsRMC-dependent Fe acquisition by comparing *OsIRO2* expression in WT and the transgenic rice plants under Fe-sufficient and Fe-deficient conditions. Overexpression of *OsRMC* and knockdown of *OsRMC* by RNAi led to enhanced and suppressed expression of *OsNAS1* in rice plants grown in Fe-sufficient medium, respectively (Fig. 8A). Fe deficiency induced upregulation of *OsNAS1* and *OsNAS2* in WT plants, and the Fe deficiency-induced upregulation of *OsNAS1* and *OsNAS2* was markedly potentiated and inhibited in the overexpression and RNAi lines, respectively. The expression level of *OsDMAS1* in the OE3 line was marginally higher than in WT plants under Fe-sufficient conditions, while *OsDMAS1* transcripts in the two RNAi lines (Ril and Ri4) were substantially lower than in WT plants under Fe-sufficient conditions (Fig. 8C). Exposure of WT plants to Fe-deficient medium led to upregulated expression of *OsDMAS1*, and the upregulation was enhanced and suppressed by overexpression and RNAi of *OsRMC*, respectively (Fig. 8C). As a consequence, transcript levels of *OsDMAS1* in the overexpression and RNAi lines were higher and lower than in WT under Fe-deficient conditions. A similar expression pattern of *OsNAAT1*, *O*s*IRT1*, *OsYSL15*, and *OsIRO2* was found in overexpression and RNAi lines such that overexpression and knockdown of *OsRMC* led to greater and less expression of *O*s*IRT1*, *OsYSL15*, and *OsIRO2*, respectively, and the effect was more pronounced under Fe-deficient conditions than under Fe-sufficient conditions (Fig. 8D–G).

## Discussion

Fe deficiency is one of the limiting factors affecting production and nutritional quality of crops due to low solubility of Fe(III)-oxides that often occur in soils, especially in calcareous soils (Guerinot and Yi, 1994). In addition to Fe deficiency in plants, more than 2 billion people have been reported to be at risk of Fe deficiency-induced anaemia worldwide (WHO, 2007). Given that plants are a primary source of Fe nutrition for humans, understanding the mechanisms by which plants efficiently acquire Fe from soil, particularly under Fe-deficient conditions, could help to alleviate Fe malnutrition of humans. Rice is a major crop and primary source of food in the majority of Asia regions (Khush, 2005). Therefore, identification of key regulators that contribute to greater accumulation of Fe in rice would be of significance for improvement of Fe nuitrition in food by breeding rice cultivars with a high Fe nutritional quality.

There are reports demonstrating that Fe-fortified grains can be obtained by molecular manipulation of genes associated with Fe mobilization and transport in plants (Kobayashi and Nishizawa, 2012). For example, an elevated Fe level in rice has been achieved by increasing the amount of metal chelators (Takahashi *et al.*, 2001; Lee *et al.*, 2009). Overexpression of specific transporters involved in absorption and translocation of Fe has also been used to enhance Fe content in plants (Lee and An, 2009; Lee *et al.*, 2009; Ishimaru *et al.*, 2010). In addition, manipulation of central transcription factors involved in regulation of the Fe-deficiency response can also improve rice growth under Fe-deficient conditions (Kobayashi *et al.*, 2007; Ogo *et al.*, 2007; Ogo *et al.*, 2008). However, the signalling networks responsible for Fe homeostasis remain largely to be dissected. In the present study, we demonstrated that a gene encoding a receptor-like protein, *OsRMC*, plays an important role in the regulation of Fe acquisition in rice. More specifically, we found that overexpression of *OsRMC* made rice seedlings more tolerant to Fe deficiency than WT rice plants, while RNAi knockdown of *OsRMC* rendered the seedling growth more sensitive to Fe deficiency. Most importantly, our results revealed that, in addition to improving the growth of overexpressing rice plants under Fe-deficient conditions, overexpression of *OsRMC* also led to enhanced accumulation of Fe and Zn in mature seeds under Fe-sufficient conditions. These findings highlight a novel function of *OsRMC* in the regulation of mineral nutrients in both plants and seeds. To the best of our knowledge, this is the first report demonstrating the involvement of receptor-like proteins in the control of mineral nutrient acquisition in higher plants.

To elucidate the mechanism underlying the efficient acquisition of Fe in transgenic rice plants overexpressing *OsRMC* under Fe-deficient conditions, physiological processes associated with MA synthesis and IRT-mediated Fe^2+^ transport were studied at the transcriptional level. Our results revealed that expression levels of *OsRMC* were positively correlated with expression of genes (*OsNAS1*, *OsNAS2*, *OsNAAT1*, *OsDMAS1*, and *OsYSL15*) involved in MA synthesis and uptake of MA-Fe^3+^, particularly under Fe-deficient conditions (Fig. 8). The upregulation of these genes would allow the transgenic plants overexpressing *OsRMC* to have more effective exudation of MAs from rice roots, which in turn act as phytosiderophores to chelate Fe^3+^ in the rhizosphere, thus facilitating Fe mobilization. *OsIRO2* is an Fe deficiency-inducible basic helix–loop–helix (bHLH) transcription factor and is responsible for regulating the genes involved in Fe homeostasis in rice, including *OsNAS1*, *OsNAS2*, *OsNAAT1*, *OsDMAS1*, and *OsYSL15*. Here, we showed that the transcriptional level of *OsIRO2* was also upregulated by overexpression of *OsRMC*, indicating that *OsRMC* is a potential upstream target of *OsIRO2*. In addition to enhanced biosynthesis of MAs, our results also showed that overexpression of *OsRMC* upregulated expression of the Fe^2+^ transporter *OsIRT1*, particularly under Fe-deficient conditions. As *OsIRT1*-mediated Fe^2+^ transport is involved in Fe^2+^ uptake in rice (Ishimaru *et al.*, 2006), enhanced expression of *OsIRT1* under Fe-deficient conditions would make the rice plants more efficient at taking up Fe^2+^ in the Fe-deficient medium, thus contributing to the observed high Fe concentrations in both shoots and roots.

Previous studies have shown that RNAi knockdown of *OsRMC* alters root development (Jiang *et al.*, 2007) and confers enhanced tolerance to salt stress (Zhang *et al.*, 2009). The two RNAi lines (Ri1 and Ri4) used in the present study displayed shorter primary roots and fewer adventitious roots than WT plants (Jiang *et al.*, 2007). Our results showing that total root length of the two RNAi lines was less than that of WT plants grown in Fe-sufficient conditions (Fig. 5A) are consistent with the results of Jiang *et al.* (2007). Torabi *et al.* (2009) showed that expression of *OsRMC* is suppressed in response to P deficiency. In contrast, we detected a higher expression level of *OsRMC* in response to P deficiency (Fig. S1). We do not have explanation for the difference between low P-induced expression of *OsRMC* in our studies and those reported by Torabi *et al.* (2009). We speculate that the differences may be due to the rice genotypes and the experimental protocols used in the two studies. In previous studies, *OsRMC* was found to be responsive to JA treatment, salt stress, and P_i_-starvation (Jiang *et al.*, 2007; Torabi *et al.*, 2009; Zhang *et al.*, 2009). In the present study, we demonstrated that deprivation of Fe, K, and S from the growth medium altered the expression patterns of *OsRMC*, suggesting that OsRMC has diverse roles in the control of rice growth and development, particularly by regulating physiological processes associated with nutrient acquisition. As a secreted protein, OsRMC can function as a ligand for receptor-like kinases (Chen 2001; Jiang *et al.* 2007; Serra *et al.* 2013). Therefore, we speculate that OsRMC may regulate the Fe-deficiency response and root development by interacting with some receptor-like kinases.

Another important finding in the present study was that overexpression and suppression of *OsRMC* significantly enhanced and reduced Fe and Zn content, respectively, in mature seeds under normal growth conditions. It has been reported that overexpression of *OsIRT1* in rice plants leads to elevated Fe and Zn level in the mature seeds (Lee and An, 2009). Constitutive overexpression of *OsNAS* genes also results in increases in the content of Fe and Zn in rice grains (Lee *et al.*, 2009; Masuda *et al.*, 2009; Johnson *et al.*, 2011). In addition to increased Fe content in shoots, overexpression of *OsIRO2* conferred greater accumulation of Fe in seeds than WT rice plants when grown on calcareous soil (Ogo *et al.*, 2011). Therefore, it is conceivable that the increased concentrations of Fe and Zn in *OsRMC*-overexpressing grains of the OE3 line may result from upregulation of the *OsIRT1*, *OsNAS1*, *OsNAS2*, and *OsIRO2* genes.

In addition to changes in physiological processes, plants can also modify their root morphological traits to maximize their Fe acquisition by increasing the root surface area (Guerinot and Yi, 1994; López-Bucio *et al.*, 2003). Examples include increased formation and branching of root hairs, root-tip swelling, and enhanced lateral root formation (Schmidt, 2002; Müller and Schmidt, 2004). In the present study, we found that transgenic rice plants with overexpression of *OsRMC* exhibited greater root systems than WT rice plants under Fe-deficient conditions, such that the total root length and root surface area in the OE3 line were greater than those in WT rice plants (Figs 5 and 6). In contrast, the total root length and surface area in the transgenic rice plants with underexpression of *OsRMC* by RNAi were smaller than those in WT rice plants in Fe-deficient medium. The increased root systems in the *OsRMC-*overexpressing line would facilitate exudation of Fe phytosiderophores and uptake of Fe due to a greater root surface area, thus making the overexpressing line more efficient at mobilization and uptake of Fe by the roots in Fe-deficient medium.

JA is an oxylipin-based plant hormone originating from polyunsaturated fatty acids that acts in response to both developmental and environmental stimuli. A recent study reported that jasmonate may act as an inhibitor to fine-tune Fe-deficiency responses, but that it is not involved in the systemic downregulation of Fe-deficiency responses in the roots of *Arabidopsis* (Maurer *et al.*, 2011). RNAi knockdown of *OsRMC* led to altered root development and coiling, which are mediated by JA signalling in rice (Jiang *et al.*, 2007). In the present study, we found that *OsRMC* played a role in adaptation of rice plants to Fe deficiency. Therefore, future work to investigate the role of JA in *OsRMC*-mediated Fe-deficiency responses is warranted.

In summary, our results demonstrated that *OsRMC* is likely to play a significant role in the regulation of Fe acquisition by upregulating biosynthesis of MAs, Fe-MA transport, IRT1-dependent Fe transport, and root development in rice under Fe-deficient conditions. In addition, we found that overexpression of *OsRMC* in rice plants resulted in greater accumulation of Fe in mature rice seeds. This observation has important implications in molecular manipulation of Fe contents in seeds. The enhanced mobilization and transport of Fe and increased root surface area due to greater root growth and development in transgenic rice plants overexpressing *OsRMC* may underpin the observed greater Fe use efficiency under Fe-deficient conditions. These novel findings will be valuable for dissecting the signalling networks associated with the response of plants to Fe deficiency in general and rice plants in particular.

## Supplementary Material

Supplementary Data

## Abbreviations:

CHL: chlorophyll
JA: jasmonic acid
MA: mugineic acid
RNAi: RNA interference
WT: wild type.

## References

[CIT0001] Afzal AJ Wood AJ Lightfoot DA 2008 Plant receptor-like serine threonine kinases: roles in signaling and plant defense. Molecular Plant–Microbe Interactions 21, 507–517 18393610 10.1094/MPMI-21-5-0507

[CIT0002] Bashir K Inoue H Nagasaka S Takahashi M Nakanishi H Mori S Nishizawa NK 2006 Cloning and characterization of deoxymugineic acid synthase genes from graminaceous plants. Journal of Biological Chemistry 281, 32395–32402 16926158 10.1074/jbc.M604133200

[CIT0003] Chen Z 2001 A superfamily of proteins with novel cysteine-rich repeats. Plant Physiology 126, 473–476 11402176 10.1104/pp.126.2.473 PMC1540112

[CIT0004] Curie C Briat JF 2003 Iron transport and signaling in plants. Annual Review of Plant Biology 54, 183–206 10.1146/annurev.arplant.54.031902.135018 14509968

[CIT0005] Eide D Broderius M Fett J Guerinot ML 1996 A novel iron-regulated metal transporter from plants identified by functional expression in yeast. Proceedings of the National Academy of Sciences, USA 93, 5624–5628 10.1073/pnas.93.11.5624 PMC39298 8643627

[CIT0006] Guerinot ML Yi Y 1994 Iron: nutritious, noxious, and not readily available. Plant Physiology 104, 815–826 12232127 10.1104/pp.104.3.815 PMC160677

[CIT0007] Hardie D 1999 Plant protein serine/threonine kinases: classification and functions. Annual Review of Plant Biology 50, 97–131 10.1146/annurev.arplant.50.1.97 15012205

[CIT0008] Hell R Stephan UW 2003 Iron uptake, trafficking and homeostasis in plants. Planta 216, 541–551 12569395 10.1007/s00425-002-0920-4

[CIT0009] Inoue H Higuchi K Takahashi M Nakanishi H Mori S Nishizawa NK 2003 Three rice nicotianamine synthase genes, *OsNAS1*, *OsNAS2*, and *OsNAS3* are expressed in cells involved in long-distance transport of iron and differentially regulated by iron. The Plant Journal 36, 366–381 14617093 10.1046/j.1365-313x.2003.01878.x

[CIT0010] Inoue H Kobayashi T Nozoye T Takahashi M Kakei Y Suzuki K Nakazono M Nakanishi H Mori S Nishizawa NK 2009 Rice OsYSL15 is an iron-regulated iron (III)-deoxymugineic acid transporter expressed in the roots and is essential for iron uptake in early growth of the seedlings. Journal of Biological Chemistry 284, 3470–3479 19049971 10.1074/jbc.M806042200

[CIT0011] Inoue H Takahashi M Kobayashi T Suzuki M Nakanishi H Mori S Nishizawa NK 2008 Identification and localisation of the rice nicotianamine aminotransferase gene *OsNAAT1* expression suggests the site of phytosiderophore synthesis in rice. Plant Molecular Biology 66, 193–203 18034312 10.1007/s11103-007-9262-8

[CIT0012] Ishimaru Y Masuda H Bashir K Inoue H Tsukamoto T Takahashi M Nakanishi H Aoki N Hirose T Ohsugi R 2010 Rice metal nicotianamine transporter, OsYSL2, is required for the long distance transport of iron and manganese. The Plant Journal 62, 379–390 20128878 10.1111/j.1365-313X.2010.04158.x

[CIT0013] Ishimaru Y Suzuki M Tsukamoto T Suzuki K Nakazono M Kobayashi T Wada Y Watanabe S Matsuhashi S Takahashi M 2006 Rice plants take up iron as an Fe^3+^-phytosiderophore and as Fe^2+^ . The Plant Journal 45, 335–346 16412081 10.1111/j.1365-313X.2005.02624.x

[CIT0014] Jiang J Li J Xu Y Han Y Bai Y Zhou G Lou Y Xu Z Chong K 2007 RNAi knockdown of *Oryza sativa root meander curling* gene led to altered root development and coiling which were mediated by jasmonic acid signalling in rice. Plant, Cell & Environment 30, 690–699 10.1111/j.1365-3040.2007.01663.x 17470145

[CIT0015] Johnson AAT Kyriacou B Callahan DL Carruthers L Stangoulis J Lombi E Tester M 2011 Constitutive overexpression of the *OsNAS* gene family reveals single-gene strategies for effective iron- and zinc-biofortification of rice endosperm. PLoS One 6, e24476 21915334 10.1371/journal.pone.0024476 PMC3167849

[CIT0016] Khush GS 2005 What it will take to feed 5.0 billion rice consumers in 2030. Plant Molecular Biology 59, 1–6 16217597 10.1007/s11103-005-2159-5

[CIT0017] Kobayashi T Itai RN Aung MS Senoura T Nakanishi H Nishizawa NK 2012 The rice transcription factor IDEF1 directly binds to iron and other divalent metals for sensing cellular iron status. The Plant Journal 69, 81–91 21880076 10.1111/j.1365-313X.2011.04772.x

[CIT0018] Kobayashi T Itai RN Ogo Y Kakei Y Nakanishi H Takahashi M Nishizawa NK 2009 The rice transcription factor IDEF1 is essential for the early response to iron deficiency, and induces vegetative expression of late embryogenesis abundant genes. The Plant Journal 60, 948–961 19737364 10.1111/j.1365-313X.2009.04015.x

[CIT0019] Kobayashi T Nishizawa NK 2012 Iron uptake, translocation, and regulation in higher plants. Annual Review of Plant Biology 63, 131–152 10.1146/annurev-arplant-042811-105522 22404471

[CIT0020] Kobayashi T Ogo Y Itai RN Nakanishi H Takahashi M Mori S Nishizawa NK 2007 The transcription factor IDEF1 regulates the response to and tolerance of iron deficiency in plants. Proceedings of the National Academy of Sciences, USA 104, 19150–19155 10.1073/pnas.0707010104 PMC2141923 18025467

[CIT0021] Lee S An G 2009 Over-expression of OsIRT1 leads to increased iron and zinc accumulations in rice. Plant, Cell & Environment 32, 408–416 10.1111/j.1365-3040.2009.01935.x 19183299

[CIT0022] Lee S Chiecko JC Kim SA Walker EL Lee Y Guerinot ML An G 2009 Disruption of OsYSL15 leads to iron inefficiency in rice plants. Plant Physiology 150, 786–800 19376836 10.1104/pp.109.135418 PMC2689993

[CIT0023] Lee S Jeon JS An G 2012 Iron homeostasis and fortification in rice. Journal of Plant Biology 55, 261–267

[CIT0024] Lee S Jeon US Lee SJ Kim YK Persson DP Husted S Schjørring JK Kakei Y Masuda H Nishizawa NK 2009 Iron fortification of rice seeds through activation of the nicotianamine synthase gene. Proceedings of the National Academy of Sciences, USA 106, 22014–22019 10.1073/pnas.0910950106 PMC2799860 20080803

[CIT0025] López-Bucio J Cruz-Ramírez A Herrera-Estrella L 2003 The role of nutrient availability in regulating root architecture. Current Opinion in Plant Biology 6, 280–287 12753979 10.1016/s1369-5266(03)00035-9

[CIT0026] Marschner H Römheld V Kissel M 1986 Different strategies in higher plants in mobilization and uptake of iron. Journal of Plant Nutrition 9, 695–713

[CIT0027] Masuda H Usuda K Kobayashi T Ishimaru Y Kakei Y Takahashi M Higuchi K Nakanishi H Mori S Nishizawa NK 2009 Overexpression of the barley nicotianamine synthase gene *HvNAS1* increases iron and zinc concentrations in rice grains. Rice 2, 155–166

[CIT0028] Maurer F Müller S Bauer P 2011 Suppression of Fe deficiency gene expression by jasmonate. Plant Physiology and Biochemistry 49, 530–536 21334215 10.1016/j.plaphy.2011.01.025

[CIT0029] Mori S 1999 Iron acquisition by plants. Current Opinion in Plant Biology 2, 250–253 10375565 10.1016/S1369-5266(99)80043-0

[CIT0030] Morris ER Walker JC 2003 Receptor-like protein kinases: the keys to response. Current Opinion in Plant Biology 6, 339–342 12873528 10.1016/s1369-5266(03)00055-4

[CIT0031] Müller M Schmidt W 2004 Environmentally induced plasticity of root hair development in Arabidopsis. Plant Physiology 134, 409–419 14730071 10.1104/pp.103.029066 PMC371035

[CIT0032] Ogo Y Itai RN Kobayashi T Aung MS Nakanishi H Nishizawa NK 2011 OsIRO2 is responsible for iron utilization in rice and improves growth and yield in calcareous soil. Plant Molecular Biology 75, 593–605 21331630 10.1007/s11103-011-9752-6

[CIT0033] Ogo Y Kobayashi T Itai RN Nakanishi H Kakei Y Takahashi M Toki S Mori S Nishizawa NK 2008 A novel NAC transcription factor, IDEF2, that recognizes the iron deficiency-responsive element 2 regulates the genes involved in iron homeostasis in plants. Journal of Biological Chemistry 283, 13407–13417 18308732 10.1074/jbc.M708732200

[CIT0034] Ogo Y Nakanishi Itai R Nakanishi H Kobayashi T Takahashi M Mori S Nishizawa NK 2007 The rice bHLH protein OsIRO2 is an essential regulator of the genes involved in Fe uptake under Fe-deficient conditions. The Plant Journal 51, 366–377 17559517 10.1111/j.1365-313X.2007.03149.x

[CIT0035] Robinson NJ Procter CM Connolly EL Guerinot ML 1999 A ferric-chelate reductase for iron uptake from soils. Nature 397, 694–697 10067892 10.1038/17800

[CIT0036] Romera FJ Alcántara E 2004 Ethylene involvement in the regulation of Fe-deficiency stress responses by Strategy I plants. Functional Plant Biology 31, 315–328 10.1071/FP03165 32688902

[CIT0037] Schmidt W 2002 Mechanisms and regulation of reduction-based iron uptake in plants. New Phytologist 141, 1–26

[CIT0038] Serra TS Figueiredo DD Cordeiro AM Almeida DM Lourenço T Abreu IA Sebastián A Fernandes L Contreras-Moreira B Oliveira MM 2013 OsRMC, a negative regulator of salt stress response in rice, is regulated by two AP2/ERF transcription factors. Plant Molecular Biology 82, 1–17 23703395 10.1007/s11103-013-0073-9

[CIT0039] Takagi S 1976 Naturally occurring iron-chelating compounds in oat-and rice-root washings. Soil Science and Plant Nutrition 22, 423–433

[CIT0040] Takagi S Nomoto K Takemoto T 1984 Physiological aspect of mugineic acid, a possible phytosiderophore of graminaceous plants. Journal of Plant Nutrition 7, 469–477

[CIT0041] Takahashi M Nakanishi H Kawasaki S Nishizawa NK Mori S 2001 Enhanced tolerance of rice to low iron availability in alkaline soils using barley nicotianamine aminotransferase genes. Nature Biotechnology 19, 466–469 10.1038/88143 11329018

[CIT0042] Torabi S Wissuwa M Heidari M Naghavi MR Gilany K Hajirezaei MR Omidi M Yazdi-Samadi B Ismail AM Salekdeh GH 2009 A comparative proteome approach to decipher the mechanism of rice adaptation to phosphorous deficiency. Proteomics 9, 159–170 19053143 10.1002/pmic.200800350

[CIT0043] Torii KU 2000 Receptor kinase activation and signal transduction in plants: an emerging picture. Current Opinion in Plant Biology 3, 361–367 11019802 10.1016/s1369-5266(00)00097-2

[CIT0044] Vert G Grotz N Dédaldéchamp F Gaymard F Guerinot ML Briat JF Curie C 2002 IRT1, an Arabidopsis transporter essential for iron uptake from the soil and for plant growth. Plant Cell 14, 1223–1233 12084823 10.1105/tpc.001388 PMC150776

[CIT0045] Wang L Ying Y Narsai R Ye L Zheng L Tian J Whelan J Shou H 2013 Identification of OsbHLH133 as a regulator of iron distribution between roots and shoots in *Oryza sativa* . Plant, Cell & Environment 36, 224–236 10.1111/j.1365-3040.2012.02569.x 22755510

[CIT0046] WHO 2007 Micronutrient deficiency: iron deficiency anaemia. World Health Organization, http://www.who.int/nutrition/topics/ida/en/index.html

[CIT0047] Zhang L Tian LH Zhao JF Song Y Zhang CJ Guo Y 2009 Identification of an apoplastic protein involved in the initial phase of salt stress response in rice root by two-dimensional electrophoresis. Plant Physiology 149, 916–928 19036832 10.1104/pp.108.131144 PMC2633861

[CIT0048] Zheng L Ying Y Wang L Wang F Whelan J Shou H 2010 Identification of a novel iron regulated basic helix–loop–helix protein involved in Fe homeostasis in *Oryza sativa* . BMC Plant Biology 10, 166 20699001 10.1186/1471-2229-10-166 PMC3017827

