# Corrigendum

**DOI:** 10.1093/jxb/ert419

**Published:** 2013-12-18

**A receptor-like protein RMC is involved in regulation of iron acquisition in rice**

**An Yang, Yansu Li, Yunyun Xu and Wen-Hao Zhang**

*Journal of Experimental Botany*, Vol. 64, pp. 5009–5020, 2013; doi: 10.1093/jxb/ert290

In the above paper the name of the third author was incorrectly spelt. The correct author list is as follows:

An Yang, Yansu Li, Yunyuan Xu and Wen-Hao Zhang

The authors would like to apologise for this error.

