# Supplementary material for: A receptor-like protein RMC is involved in regulation of iron acquisition in rice
Source: J Exp Bot. 2013 Sep 7;64(16):5009–20. doi: 10.1093/jxb/ert290 (PMC3830483; doi:10.1093/jxb/ert290)
Supplement: Supplementary Data [file supp_64_16_5009__index.html]

A receptor-like protein RMC is involved in regulation of iron acquisition in rice — A receptor-like protein RMC is involved in regulation of iron acquisition in rice — Supplementary Data 

# A receptor-like protein RMC is involved in regulation of iron acquisition in rice

## Supplementary Data

Data files

**Files in this Data Supplement:**

- Supplementary Data - Supplementary Data
