# Supplementary material for: A receptor-like protein RMC is involved in regulation of iron acquisition in rice
Source: J Exp Bot. 2013 Sep 7;64(16):5009–20. doi: 10.1093/jxb/ert290 (PMC3830483; doi:10.1093/jxb/ert290)
Supplement: Supplementary Data [file supp_ert290_jexbot104653_file001.pdf]

Title: A receptor-like protein OsRMC is involved in regulation of iron acquisition in rice

Authors: An Yang, Yansu Li, Yunyun Xu, Wen-Hao Zhang

Supplementary Data

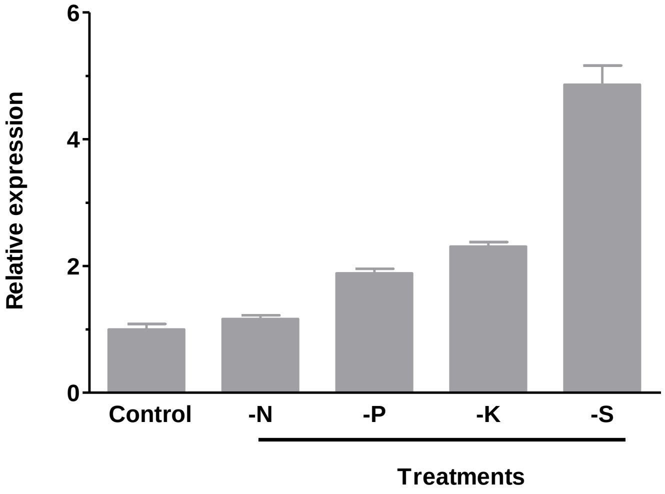

Figure S1 Transcript abundance of *OsRMC* during different nutrient deficiency treatments.

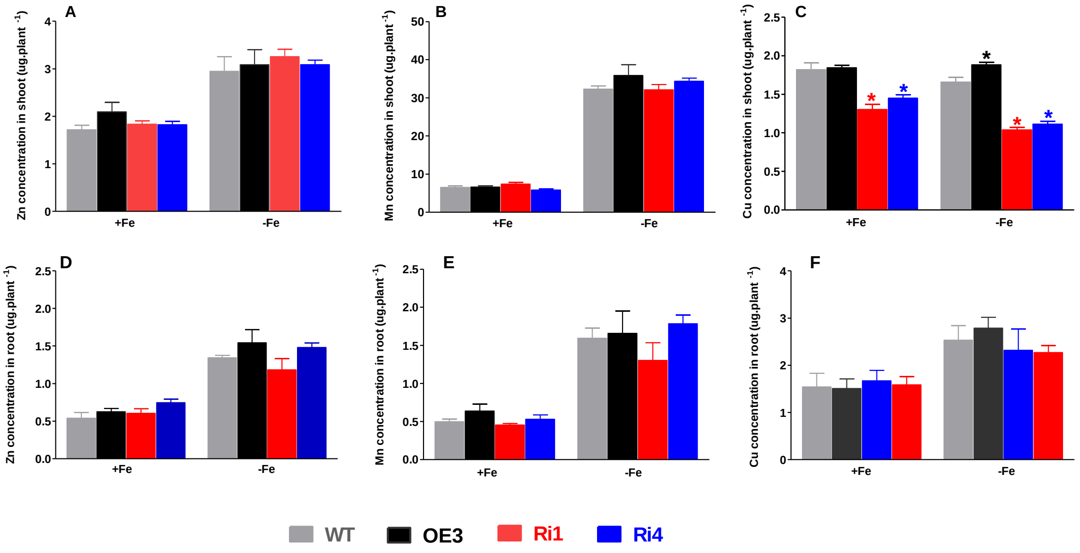

Figure S2. Quantification of metal concentration in shoots (A-C) and roots (D-F) of wild-type and transgenic plants. (A) and (D), Zn concentration (B) and (E), Mn concentration (C) and (F), Cu concentration. One-week-old seedlings were grown hydroponically for 20 days in Fe-sufficient or Fe-deficient medium, and then plants were sampled for the measurements. Significant differences from WT were determined by Student’s t-test.*P<0.05.
